# SARS‐CoV‐2 and Cancer Biology: Exploring the Mechanistic Links

**DOI:** 10.1002/cnr2.70629

**Published:** 2026-07-25

**Authors:** Andrea Orue, Alejandro Cornejo, Héctor Rafael Rangel

**Affiliations:** ^1^ Laboratorio de Biología de Tumores. Centro de Microbiología y Biología Celular Instituto Venezolano de Investigaciones Científicas. IVIC Caracas Venezuela; ^2^ Laboratorio de Virología Molecular. Centro de Microbiología y Biología Celular Instituto Venezolano de Investigaciones Científicas. IVIC Caracas Venezuela

**Keywords:** cancer, COVID‐19, immunomodulation, miRNA, oncogenesis, SARS‐CoV‐2, tumor suppressor

## Abstract

**Background:**

Five years after the emergence of SARS‐CoV‐2 and the declaration of the COVID‐19 pandemic, the long‐term implications of COVID‐19 for cancer biology remain incompletely understood. Beyond the major disruptions in cancer screening, diagnosis, and treatment observed worldwide, increasing attention has focused on whether SARS‐CoV‐2 infection and post‐acute sequelae of COVID‐19 (Long COVID) may induce persistent biological alterations relevant to tumor progression or recurrence.

**Recent Findings:**

Current evidence does not support SARS‐CoV‐2 as a classical oncogenic virus or demonstrate direct viral carcinogenesis. However, experimental, transcriptomic, and clinical studies suggest that SARS‐CoV‐2 infection can induce persistent inflammatory and immune alterations that overlap with pathways implicated in cancer biology. Among the most consistently reported findings are chronic activation of IL‐6/STAT3 and NF‐κB signaling, immune dysregulation, T‐cell exhaustion, oxidative stress, mitochondrial dysfunction, and senescence‐associated inflammatory programs. Additional proposed mechanisms include perturbation of tumor suppressor pathways, epigenetic remodeling, and microRNA alterations involving the let‐7/LIN28B/STAT3 axis. Experimental models have further suggested that inflammatory remodeling induced by respiratory viral infection may influence dormant tumor cell behavior and tissue microenvironments under defined conditions. However, many of these observations derive from in vitro systems, animal models, or association studies, and their long‐term relevance to human oncogenesis remains uncertain.

**Conclusion:**

Collectively, current evidence supports the existence of convergent biological mechanisms between SARS‐CoV‐2–induced inflammatory stress responses and pathways involved in cancer progression, rather than direct oncogenic transformation. Future longitudinal studies integrating immune profiling, inflammatory biomarkers, transcriptomic and epigenetic analyses, and clinical cancer outcomes will be essential to determine whether persistent post‐infectious alterations contribute to tumor progression, recurrence, or susceptibility in selected patient populations.

## 1 Introduction

Coronavirus disease 2019 (COVID‐19), caused by Severe Acute Respiratory Syndrome Coronavirus 2 (SARS‐CoV‐2), rapidly evolved from a localized outbreak into a global pandemic, profoundly disrupting healthcare systems worldwide [1]. Among the most severely impacted sectors was oncology care, which experienced significant interruptions in cancer screening programs, diagnostics, and therapeutic interventions during lockdown periods [2, 3, 4].

Multiple studies have reported a marked decline in new cancer diagnoses and substantial delays in treatment initiation, effects that were particularly pronounced in low‐income and middle‐income countries where healthcare systems were under unprecedented pressure [5, 6, 7, 8, 9, 10]. A systematic review encompassing 62 studies across 15 countries documented oncological treatment delays in over 75% of analyzed cases, alongside substantial reductions in cancer‐related hospitalizations [11].

Beyond healthcare disruption, patients with cancer emerged as a particularly vulnerable population during the pandemic. Individuals with malignancies exhibited increased susceptibility to SARS‐CoV‐2 infection and an approximately two‐fold higher mortality rate compared with non‐cancer patients, with outcomes varying according to tumor type, stage, and treatment status [11]. These observations underscore the existence of shared biological vulnerabilities, particularly systemic immune dysregulation and chronic inflammation [10].

In parallel, epidemiological analyses have reported an increased proportion of metastatic‐stage solid tumors diagnosed after the COVID‐19 outbreak, although the magnitude of these observations varies substantially across tumor types and geographic regions [12]. Importantly, these post‐pandemic trends should be interpreted cautiously, as delayed diagnosis, healthcare backlogs, reduced screening programs, and surveillance‐related biases likely represent major contributing factors. Consequently, future longitudinal studies assessing the long‐term consequences of the pandemic must rigorously account for these healthcare‐disruption variables in order to accurately evaluate impacts on cancer incidence, progression, and patient survival [12].

Accumulating evidence further suggests that SARS‐CoV‐2 infection may induce persistent systemic alterations extending beyond the acute phase of infection. Several mechanisms have been proposed to explain these post‐acute sequelae, including viral persistence in tissue reservoirs [11, 13, 14, 15], chronic inflammation and immune dysregulation, endothelial dysfunction, microbiota alterations, and autoimmune responses [16, 17]. In addition, some studies have associated this dysregulated immune state with reactivation of latent pathogens such as Epstein–Barr virus (EBV) and human herpesvirus 6 (HHV‐6) [18, 19].

Recent clinical and experimental studies have also suggested that SARS‐CoV‐2 infection may transiently generate microenvironmental conditions characterized by an immune‐evasive microenvironment, metabolic stress, and inflammatory signaling that resemble processes observed in cancer progression. For example, Abooali et al. described elevated structural, non‐structural, and accessory proteins that critically regulated L‐kynurenine levels and PD‐L1 upregulation within nasopharyngeal microenvironments during infection, supporting the concept of a metabolically and immunologically altered tissue niche [20]. However, whether these transient alterations can contribute to long‐term oncogenic processes remains unknown and requires prospective longitudinal investigation [20, 21].

**FIGURE 1:**
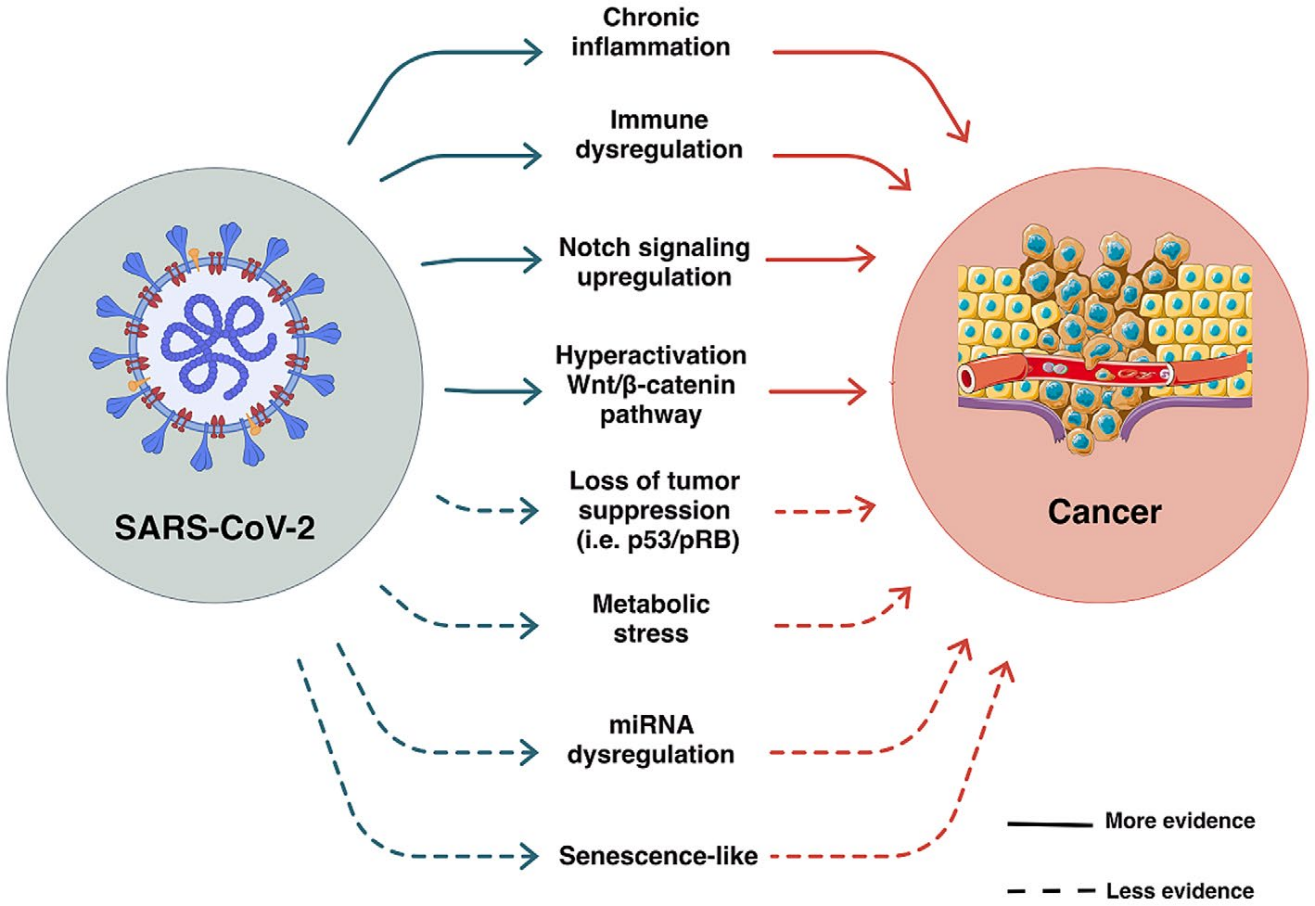
Suggested biological pathways convergence between SARS‐CoV‐2 infection and oncogenic pathways. Schematic representation of the overlapping molecular networks and cellular perturbations altered during SARS‐CoV‐2 infection and their potential contribution to cancer development. Solid arrows represent biological processes with more evidence (characterized via in vitro systems, transcriptomic analyses, or animal models), including chronic inflammation, immune dysregulation, Notch signaling upregulation, and hyperactivation of the Wnt/β‐catenin pathway. Dashed arrows indicate pathways with less evidence, reflecting speculative, indirect, or highly context‐dependent interactions, such as loss of tumor suppressor (i.e., p53/pRB), metabolic stress, miRNA dysregulation, and senescence‐like state. This conceptual framework highlights shared pathological hallmarks without implying definitive oncogenic causality, emphasizing the critical need for prospective epidemiological and longitudinal validation.

In this context, the present review explores the hypothesis that SARS‐CoV‐2 infection may interact with pathways relevant to cancer biology through indirect but convergent mechanisms involving inflammation, immune dysregulation, metabolic reprogramming, tumor suppressor pathway, and genomic stress, while emphasizing the current limitations of the available evidence and the need for long‐term clinical validation. Figure 1 summarizes the principal biological pathways proposed to connect SARS‐CoV‐2 infection with cancer‐related processes, distinguishing mechanisms supported by experimental or clinical evidence from those that remain primarily hypothetical.

## 2 Literature Review

This review represents a conceptual overview, prioritizing experimental evidence (in vitro and in vivo) and clinical reports that elucidate the molecular pathways linking SARS‐CoV‐2 and oncogenesis. We performed a structured literature search in the PubMed database using Medical Subject Headings (MeSH) and targeted keywords, including “SARS‐CoV‐2,” “tumor‐suppressor,” “cancer,” “COVID‐19,” “miRNA,” “immunomodulation.” Electronic database searches were restricted to English. Articles published between 2020 and 2026 were included.

## 3 SARS‐CoV‐2 Biology: Beyond Viral Replication

SARS‐CoV‐2 encodes 29 proteins classified as structural, non‐structural, and accessory proteins that are critical for viral replication, infectivity, and modulation of host responses [22, 23]. The structural proteins: the spike (S), nucleocapsid (N), membrane (M), and envelope (E) proteins have been extensively studied and play central roles in viral pathogenesis and as targets for therapeutics and vaccines [24, 25].

The principal function of the nucleocapsid is to protect viral RNA and to play essential roles in packaging the genome and replication; however, it also modulates host antiviral defenses by recruiting G3BP proteins to prevent stress granule formation, affecting inflammatory pathways, and promoting viral replication [25, 26]. The membrane (M) and envelope (E) proteins are involved in viral assembly/morphogenesis. The M protein shapes the viral envelope and participates in the incorporation of S and N proteins, while the E protein participates in budding and exhibits viroporin activity that can modulate the host cell environment [27, 28].

In addition to structural proteins, SARS‐CoV‐2 expresses multiple non‐structural proteins (nsps), which are involved in replication, transcription, and immune modulation. Notably, nsp1, a unique type of protein in the beta‐coronaviruses, inhibits host mRNA translation and suppresses innate immune signaling, contributing to immune evasion [29, 30]. Additionally, it has been described that this protein promotes retention in the G0/G1 phase. nsp3 and nsp15 (PLpro and Mpro, respectively) show protease activity and participate in the proteolytic steps of different polyproteins and in the virion maturation [31]. Experimental studies have proposed that some SARS‐CoV‐2 non‐structural proteins may interfere with cellular pathways associated with cell‐cycle regulation and stress responses; however, these observations derive predominantly from in vitro systems and their relevance during human infection remains uncertain [32, 33, 34]. Nevertheless, nsp6 has been associated with immune evasion through modulation of autophagy [32].

There is emerging evidence that certain accessory proteins, such as ORF3a, can activate inflammasome pathways and contribute to the increased cytokine production associated with severe COVID‐19 [35]. Additionally, a controversial hypothesis has suggested the still unconfirmed possibility that retrotranscribed SARS‐CoV‐2 sequences could contribute to persistent antigen expression and chronic inflammatory responses; however, this hypothesis remains highly debated and lacks definitive experimental confirmation [36]. However, the specific contribution of these viral proteins and the physiological relevance of these mechanisms during human infection remain to be fully established. Collectively, these observations suggest that SARS‐CoV‐2 proteins may exert broader effects on host cellular signaling beyond viral replication alone. However, the extent to which these perturbations persist or contribute to long‐term pathological consequences in vivo remains to be established.

## 4 Viral Infections and the Hallmarks of Cancer

Hallmarks of Cancer were updated in 2022 and include [37, 38, 39]: (1) sustaining proliferative signaling, (2) evading growth suppressors, (3) enabling replicative immortality, (4) activating invasion and metastasis, (5) angiogenesis, (6) resisting cell death, (7) metabolic reprogramming, (8) senescent cells, (9) immune evasion, (10) tumor‐promoting inflammation, (11) epigenetic plasticity, and (12) microbiome dysregulation [40]. Notably, viral infections (including coronaviruses) can induce reactive oxygen species (ROS) production [41], disrupt cell–cell contact inhibition [38], alter human microRNA (miRNA) networks [42], and modulate signaling pathways involved in inflammation, metabolism, and cellular stress responses, such as Wnt/β‐catenin and PI3K/AKT [43]. However, it is important to distinguish between established mechanistic observations (mainly derived from in vitro studies, animal models, and transcriptomic analyses of infected tissues) and long‐term carcinogenic outcomes, which remain unproven in the context of SARS‐CoV‐2 infection.

Among these hallmarks, the strongest evidence in SARS‐CoV‐2 infection currently supports tumor‐promoting inflammation, immune dysregulation, metabolic reprogramming, senescence‐associated alterations, and epigenetic or miRNA dysregulation, based on clinical observations, post‐mortem analyses, transcriptomic analyses, and experimental studies [11, 20, 21, 27, 34, 42]. In contrast, associations with sustained proliferative signaling, replicative immortality, invasion/metastasis, and direct carcinogenesis remain largely hypothetical and require further experimental and longitudinal validation. These shared mechanisms provide a conceptual framework to examine SARS‐CoV‐2–induced alterations in the context of cancer biology.

## 5 SARS‐CoV‐2 and Cancer: Convergent Mechanisms

Although SARS‐CoV‐2 is not currently classified as a classical oncogenic virus, accumulating evidence indicates that SARS‐CoV‐2 infection may induce persistent inflammatory, immunological, and metabolic alterations in subsets of patients with post‐acute sequelae (Long COVID) [44]. In this context, SARS‐CoV‐2 may converge on biological mechanisms with tumor‐promoting viral infections through transient but biologically relevant perturbations of inflammatory signaling, immune regulation, oxidative stress responses, and cell‐cycle control pathways [42, 43].

**FIGURE 2:**
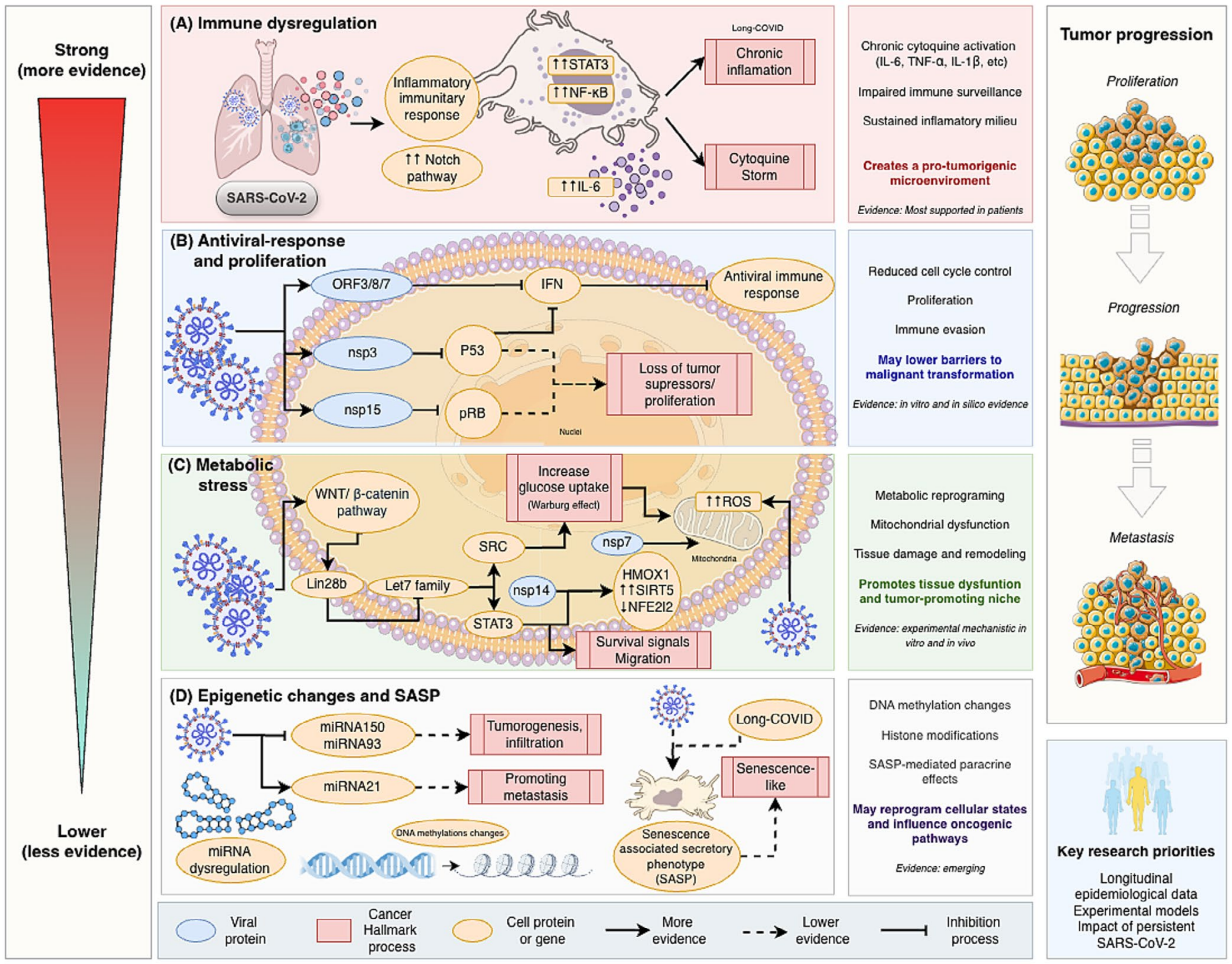
Overview of potential mechanistic links between SARS‐CoV‐2 infection, immune dysregulation, and neoplastic pathways. Conceptual mapping categorized by a validation gradient based on the strength of current scientific evidence (*y*‐axis; top: Prominent clinical/experimental observations; bottom: Preliminary or theoretical models), illustrating how viral‐induced molecular perturbations align with stages of neoplastic progression (proliferation, progression, and metastasis; right panel). (A) Immune dysregulation and chronic inflammation (high clinical evidence): Acute and post‐acute SARS‐CoV‐2 pathogenesis drives an aberrant immune response characterized by Notch pathway upregulation and the hyperactivation of the STAT3 and NF‐κB signaling axes. This sustained cascade fuels an IL‐6‐mediated pro‐inflammatory secretome (associated with post‐acute sequelae/Long COVID) and systemic cytokine release syndrome, establishing a permissive, pro‐tumorigenic microenvironment through established clinical mechanisms. (B) Antiviral response suppression and cell‐cycle deregulation (potential and theoretical pathways): viral accessory and non‐structural proteins (including ORF3, ORF6, ORF7, nsp3, and nsp15) disrupt Type‐I interferon (IFN) signaling—a well‐characterized potential mechanistic pathway. Conversely, their targeted interference with critical host tumor suppressors (p53 and pRB) and the subsequent abrogation of homeostatic checkpoints remain largely theoretical mechanisms within clinical settings, requiring further experimental validation. (C) Metabolic reprogramming and oxidative stress (potential mechanistic intersections): Viral components (e.g., nsp7 and nsp14) modulate highly conserved oncogenic networks, including the Wnt/β‐catenin axis, the Lin28/Let‐7 microRNA family, and SRC/STAT3 signaling. These cross‐talks represent potential mechanisms that induce mitochondrial dysfunction, elevated reactive oxygen species (ROS) production, and accelerated glucose uptake (the Warburg effect), driving metabolic tissue remodeling and a tumor‐supportive niche in vitro. (D) Epigenetic plasticity and cellular senescence (emerging and hypothetical links): emerging data indicate that SARS‐CoV‐2 infection alters host non‐coding RNA networks (including dysregulation of miRNA‐150, miRNA‐93, and miRNA‐21) and induces DNA methylation changes or telomere shortening. These alterations promote a Senescence‐Associated Secretory Phenotype (SASP) linked to chronic host dysregulation; however, its capacity to definitively facilitate epigenetic plasticity, cellular reprogramming, or *de novo* transformation represents a hypothetical link that remains unproven in patients. Right Inset: Delineates definitive research priorities, emphasizing the mandatory epidemiological and long‐term longitudinal validation required to confirm these hypothetical and potential mechanistic intersections.

Several aspects warrant further investigation: persistent viral reservoirs, Long‐COVID, repeated reinfections, and reactivation of latent viruses. At present, there is no direct epidemiological evidence demonstrating that individuals with Long COVID exhibit different cancer incidence or outcomes compared to fully recovered individuals [42]. Therefore, Long COVID should be considered a potential biological context of sustained host dysregulation rather than a confirmed oncogenic risk state [43] (Figure 2).

Several hypotheses have been proposed to explain post‐acute sequelae, including persistent viral reservoirs in tissues [13], immune dysregulation [14, 20], and reactivation of latent viruses such as EBV and human herpesvirus 6 (HHV‐6) [13, 17, 18, 45]. Collectively, these processes may contribute to a sustained inflammatory and immunomodulatory state characterized by cytokine imbalance, impaired interferon signaling, microbiome disruption [18], endothelial dysfunction, and autoimmunity [46, 47] (See Figure 2A).

Inducing severe inflammatory states: SARS‐CoV‐2 infection can lead to the release of pro‐inflammatory cytokines and a reduced interferon response, creating a microenvironment conducive to viral spread and potential oncogenic transformation [48], associated with excessive cytokine release, Th17 skewing, IL‐6 overproduction, and activation of Wnt/β‐catenin‐Notch signaling, which are established drivers of tumor‐promoting inflammation [49, 50, 51] (See Figure 2A). These mechanisms overlap with pathways implicated in tumor promotion, including chronic inflammation and impaired immune surveillance; a causal relationship between SARS‐CoV‐2 infection (including Long COVID) and cancer development has not been established and requires long‐term longitudinal investigation.

According to current evidence and potential relevance to human cancer biology, the mechanism we would rank them approximately as follows: (1) persistent inflammation and immune dysregulation, including chronic IL‐6/STAT3 and NF‐κB activation, cytokine imbalance, T‐cell exhaustion, and impaired immune surveillance, as these processes are consistently documented in acute severe COVID‐19 and subsets of Long COVID patients [14, 20]. (2) Senescence‐like cellular stress responses and metabolic dysregulation, including oxidative stress, mitochondrial dysfunction, and SASP‐associated inflammatory signaling, which are supported by experimental studies and some human tissue analyses [51]. (3) Epigenetic and transcriptomic alterations, including miRNA dysregulation and changes in inflammatory or stress‐response pathways, although their persistence and biological consequences remain incompletely understood. (4) Perturbation of tumor suppressor pathways such as p53 and pRB, which are currently supported primarily by mechanistic and in vitro studies, with limited direct in vivo validation in human infection. (5) Direct oncogenic or transformation‐like mechanisms, including stable genomic instability or persistent virus‐driven carcinogenic processes, which remain highly speculative and currently lack convincing clinical evidence.

## 6 Immune Dysregulation and Cellular Senescence as Convergent Links Between SARS‐CoV‐2 Infection and Cancer

SARS‐CoV‐2 infection induces cellular stress responses that may persist beyond the acute phase and contribute to prolonged tissue dysfunction [52]. Among these responses, senescence‐like cellular programs have emerged as potential mediators linking viral infection, chronic inflammation, and immune dysregulation [53]. Like other RNA viruses, SARS‐CoV‐2 can induce senescence in infected cells and also affect neighboring non‐infected cells (bystander cells) through paracrine signaling, thereby amplifying local inflammatory responses and contributing to the establishment of a senescence‐associated secretory phenotype (SASP) characterized by sustained secretion of pro‐inflammatory cytokines, chemokines, matrix‐remodeling enzymes, and pro‐coagulatory factors [51]. These SASP components amplify local and systemic inflammation and are thought to contribute to the cytokine storm observed in severe COVID‐19 (See Figure 2D).

Classical cellular senescence refers to a stable cell‐cycle arrest triggered by stressors such as DNA damage, oxidative stress, mitochondrial dysfunction, or metabolic imbalance [48]. Senescent cells acquire a senescence‐associated secretory phenotype (SASP), characterized by secretion of pro‐inflammatory cytokines, chemokines, growth factors, and matrix‐remodeling enzymes [51]. These mediators can act in paracrine and autocrine fashions, affecting neighboring non‐infected (“bystander”) cells and amplifying local inflammatory responses [54].

Importantly, many SARS‐CoV‐2 target cells within the respiratory tract are terminally differentiated epithelial populations with limited proliferative capacity. In this context, the term senescence in COVID‐19 literature is often used operationally to describe senescence‐like transcriptional and inflammatory stress programs rather than canonical irreversible proliferative arrest [51]. Experimental and post‐mortem studies have reported increased expression of senescence‐associated markers in infected lung tissue, supporting the existence of virus‐associated senescence‐like responses during severe disease [55].

At the molecular level, these responses are regulated primarily through the p53/p21 and p16/RB tumor suppressor pathways [52]. SARS‐CoV‐2 infection has been associated with increased oxidative stress, mitochondrial dysfunction, impaired autophagy, and activation of DNA damage response pathways, including ataxia telangiectasia and Rad3‐related (ATR) signaling, a central mediator of replicative stress and genomic damage responses [56]. Elevated reactive oxygen species (ROS) may further contribute to inflammatory amplification, mitochondrial dysfunction, and cellular stress; however, the persistence, tissue specificity, and biological consequences of these responses in vivo remain under investigation.

Post‐mortem and clinical studies have also reported telomere shortening and epigenetic alterations in subsets of COVID‐19 patients [57, 58]. Several studies also report accelerated biological aging and telomere attrition in post COVID‐19 patients, particularly in individuals younger than 60 years, although these findings remain heterogeneous and may be reversible in some cases [58]. Nevertheless, telomere attrition is a non‐specific marker influenced by aging, systemic inflammation, oxidative stress, and severe illness. Therefore, these findings should be interpreted cautiously, as similar alterations may occur in other infectious or inflammatory conditions and are not necessarily unique to SARS‐CoV‐2 infection.

Senescence‐driven inflammation directly intersects with immune dysregulation, a central feature of both acute and post‐acute SARS‐CoV‐2 infection. The immune response to SARS‐CoV‐2 is biphasic, beginning with an innate phase mediated by pattern recognition receptors (PRRs) that detect viral RNA and trigger type I interferon (IFN‐I) production [48]. Timely IFN‐I signaling is essential for viral control. However, delayed or blunted IFN responses permit viral persistence and excessive recruitment of inflammatory myeloid cells, including monocytes, macrophages, and neutrophils [48]. This dysregulation promotes a hyperinflammatory state characterized by elevated IL‐6, TNF‐α, IL‐1β, IFN‐γ, and CXCL10 [59]. Sustained activation of NF‐κB and STAT3 signaling contributes to feed‐forward inflammatory amplification resembling pathways active in chronic inflammatory states and the tumor microenvironment [59, 60].

The adaptive immune compartment is likewise profoundly affected. Although SARS‐CoV‐2 specific CD4^+^ and CD8^+^ T cell responses are detectable in most infected individuals, severe disease is associated with marked lymphopenia, T cell exhaustion, and reduced IFN‐γ production [20, 61]. Expansion of immunosuppressive myeloid populations, including myeloid‐derived suppressor cells (MDSCs) and low‐density neutrophils (LDNs), further suppresses cytotoxic T cell function and mirrors immune landscapes observed in advanced cancers [61, 62]. These alterations partially resemble immune dysfunction patterns observed in advanced malignancies and may impair immune surveillance [63], although direct implications for long‐term oncogenesis remain unproven.

Persistent immune perturbations have been documented in subsets of post‐acute COVID‐19 patients for months after infection, including sustained inflammatory signaling, mitochondrial stress, aberrant Th17 responses, and prolonged myeloid activation [14]. Studies such as Phetsouphanh et al. reported immunological abnormalities persisting for up to 8 months following mild‐to‐moderate infection, while other cohorts suggest persistence in subsets of symptomatic patients for up to 24 months. However, the magnitude and clinical significance of these alterations vary substantially between studies and patient populations [14].

Chronic inflammation and immune dysfunction converge at the level of post‐transcriptional regulation. Persistent activation of IL‐6/STAT3 and NF‐κB signaling induces expression of Lin28B, leading to repression of the tumor‐suppressive let‐7 microRNA family [64]. Loss of let‐7 derepresses oncogenic targets including STAT3, HMGA2, RAS, and components of the Wnt/β‐catenin pathway, reinforcing cellular plasticity, stemness, metabolic reprogramming, and resistance to immune‐mediated clearance. This let‐7–STAT3–Wnt axis represents a shared molecular framework linking SARS‐CoV‐2‐induced immune dysregulation with pathways central to cancer initiation and progression [65] (See Figure 2C).

Experimental studies in murine models have further suggested that respiratory viral infections may influence tumor cell behavior. In particular, SARS‐CoV‐2–driven inflammatory signaling disrupted dormancy of disseminated carcinoma cells in the lung through IL‐6–dependent mechanisms, promoting metastatic outgrowth under inflammatory conditions [63]. While these findings provide mechanistic support for the concept that respiratory infections may alter tumor microenvironment dynamics, they are currently limited to preclinical systems and should not be interpreted as evidence of direct metastatic reactivation in humans.

Together, senescence‐like responses and immune dysregulation emerge as interconnected biological processes linking SARS‐CoV‐2 infection with pathways relevant to cancer biology. However, current evidence primarily supports overlap at the level of inflammatory signaling networks, immune suppression, metabolic stress, senescence, and hypothetical tumor suppressor dysfunction, rather than direct oncogenic transformation. Whether these persistent post‐infectious alterations contribute meaningfully to cancer initiation, progression, or recurrence in susceptible individuals remains an open question requiring longitudinal clinical investigation.

## 7 SARS‐CoV‐2 and Potential Disruption of Tumor Suppressor Pathways

Several studies have suggested that SARS‐CoV‐2 infection may converge on host tumor suppressor pathways that are also targeted by established oncogenic viruses such as EBV, hepatitis B virus (HBV), and human papillomavirus (HPV), particularly those involving p53 and retinoblastoma protein (pRB) signaling [66, 67] (Figure 2B). Nevertheless, unlike classical oncoviruses, SARS‐CoV‐2 does not appear to encode dedicated transforming oncoproteins. Rather, perturbation of these pathways is hypothesized to occur indirectly through inflammatory signaling, oxidative stress, metabolic stress, and virus‐induced host responses capable of modulating protein stability, transcriptional regulation, DNA damage signaling, and cell‐cycle control [41, 66, 67].

### 7.1 p53 Tumor Suppressor

Tumor protein 53 (p53) is a central regulator of genomic integrity, cell‐cycle arrest, apoptosis, senescence, and immune surveillance [68]. As one of the most important tumor suppressors, p53 is functionally altered in more than half of human cancers [69]. Coronaviruses reduce endogenous p53 levels in infected cells by promoting proteasomal degradation, suggesting that modulation of p53‐associated pathways may contribute to viral pathogenesis [70].

Experimental studies indicate that SARS‐CoV‐2 may interfere with p53‐related signaling through multiple mechanisms. Proposed interactions involving viral non‐structural proteins and prohibitin complexes (PHB1/2) have been associated with mitochondrial dysfunction, oxidative stress, and altered stress‐response signaling [66, 71]. In addition, the SARS‐CoV‐2 non‐structural protein 3 (nsp3) has been proposed to promote p53 degradation through ubiquitin ligase pathways involving RCHY1 and MDM2 [41, 67]. However, most of these observations derive from in vitro or mechanistic studies, and their physiological relevance during human infection remains incompletely established.

Transcriptomic and bioinformatic analyses have also identified alterations in p53‐associated pathways in severe COVID‐19 and some Long COVID cohorts, including pathways involved in apoptosis, DNA damage responses, inflammation, and immune regulation [72, 73]. Moreover, persistent circulating SARS‐CoV‐2 antigens, including spike protein, have been detected in subsets of Long COVID patients months after acute infection, raising the possibility that virus‐associated inflammatory and stress‐related signaling may extend beyond the acute phase [11, 15]. Nevertheless, there is currently no clinical evidence demonstrating stable p53 genetic inactivation or persistent functional suppression comparable to that observed in p53‐mutated cancers. Therefore, the potential oncogenic implications of these findings remain hypothetical and require long‐term mechanistic and longitudinal clinical validation.

### 7.2 Retinoblastoma Protein (pRB)

The pRB regulates cell‐cycle progression and epigenetic modifications, and loss of its function is associated with uncontrolled proliferation and cancer [74]. Studies in SARS‐CoV‐1 demonstrated that the viral endoribonuclease non‐structural protein 15 (nsp15) interacts with pRB through an LXCXE motif, a conserved sequence involved in pRB binding and inactivation [75]. This interaction promoted pRB nuclear export, ubiquitination, and proteasomal degradation, while cells expressing SARS‐CoV nsp15 exhibited loss of contact inhibition and hyperproliferative phenotypes [75]. Because SARS‐CoV‐2 nsp15 is highly conserved and retains an LXCXE‐like motif, a similar interaction has been hypothesized, although direct functional confirmation during SARS‐CoV‐2 infection remains limited [42, 75]. These observations suggest that coronavirus‐mediated perturbation of pRB signaling may contribute to dysregulated cell cycle signaling under certain experimental conditions; however, whether these effects occur in vivo during SARS‐CoV‐2 infection remains to be established.

## 8 Metabolic Reprogramming and Cell Death Pathways: COVID‐19 and Cancer Metabolic Relationships

Viral replication and tumor growth share common metabolic requirements, including increased anabolic metabolism, redox adaptation, and iron handling [76, 77]. In this context, SARS‐CoV‐2 infection has been reported to induce metabolic rewiring that partially resembles cancer‐associated metabolic phenotypes [78] (See Figure 2C). The transcription factor c‐Myc is a central regulator of these processes, controlling glycolysis, nucleotide biosynthesis, and iron metabolism [78].

Under physiological conditions, c‐Myc activity is tightly regulated and negatively controlled by p53‐dependent checkpoints [78, 79, 80]. Therefore, disruption of p53‐associated signaling during SARS‐CoV‐2 infection could contribute indirectly to metabolic dysregulation. However, whether c‐Myc activation observed in experimental systems reflects direct viral protein activity or secondary inflammatory and metabolic stress responses remains unresolved.

Several viral proteins have been implicated in modulating host metabolic and stress‐response pathways in in vitro models or protein overexpression systems. For example, ORF7b has been reported to induce apoptosis and ferroptosis in lung epithelial cells via c‐Myc‐associated signaling and iron‐dependent lipid peroxidation [79]. Ferroptosis is a regulated, iron‐dependent form of non‐apoptotic cell death increasingly recognized in cancer biology and therapy resistance [79]. However, most mechanistic evidence in SARS‐CoV‐2 infection derives from experimental models, whereas in vivo validation in infected human tissues remains limited.

Similarly, ORF3a, ORF6, and ORF8 have been shown to interfere with host antiviral and stress‐response pathways in experimental systems, including modulation of caspase activation, interferon signaling, and NRF2‐dependent antioxidant responses [81, 82]. Additional non‐structural proteins, including nsp7 and nsp14, have been implicated in mitochondrial dysfunction, redox imbalance [43], and modulation of antioxidant regulators such as HMOX1 and SIRT5, although these findings are largely derived from experimental interaction studies rather than direct observations in infected human tissues [43, 83].

Importantly, post‐mortem analyses of lung tissue from patients with fatal COVID‐19 have reported molecular signatures consistent with oxidative stress, lipid peroxidation, and ferroptosis‐associated injury, including increased transferrin receptor 1 (TfR1) expression [84]. These findings provide ex vivo support for the activation of stress‐related cell death pathways during severe disease. Collectively, these observations suggest that SARS‐CoV‐2 infection may engage oxidative stress, metabolic rewiring, and regulated cell death pathways that overlap with mechanisms implicated in cancer biology. However, the majority of evidence currently derives from experimental or post‐mortem studies, and the extent to which these processes contribute to long‐term genomic instability or tumor initiation in vivo remains speculative and requires longitudinal validation [9, 78, 84].

## 9 Wnt/β‐Catenin Signaling in SARS‐CoV‐2 Infection and Cancer

The Wnt/β‐catenin pathway is a highly conserved signaling cascade involved in embryogenesis, tissue regeneration, stem cell maintenance, and cellular plasticity. Aberrant activation of canonical Wnt signaling is implicated in multiple malignancies, particularly colorectal carcinoma [85, 86]. Canonical Wnt signaling stabilizes β‐catenin and promotes transcription of genes involved in proliferation, survival, stemness, and metabolism, including c‐MYC, whereas non‐canonical Wnt pathways regulate cell polarity and motility [87].

Modulation of Wnt/β‐catenin signaling has been reported during SARS‐CoV‐2 infection in experimental infection systems, transcriptomic analyses, and patient‐derived samples. These alterations have been observed in respiratory epithelial cells, immune cells, and neural‐derived cells [88]. However, the direction and magnitude of pathway activation appear to depend on the cellular context and inflammatory state. Importantly, it remains unclear whether these changes reflect direct virus‐host interactions or secondary responses to cytokine signaling, hypoxia, oxidative stress, and tissue injury [87, 88, 89, 90].

Experimental studies using viral proteins have provided mechanistic support for the ability of SARS‐CoV‐2 related components to engage Wnt‐associated pathways under defined conditions. In cellular and animal models, spike protein exposure induced senescence‐like cellular programs through cell division cycle protein 42 (CDC42) upregulation (which plays a pivotal role in cytoskeleton organization, cell polarity, cell growth, and carcinogenesis) and subsequent activation of Wnt/β‐catenin signaling, contributing to inflammatory and lung injury phenotypes [88]. Similarly, alterations in Wnt/β‐catenin pathway components have been reported in SARS‐CoV‐2–infected astrocytes in vitro [90]. However, these observations derive primarily from experimental systems, and their relevance during human infection remains to be fully established.

Beyond its role in tissue remodeling, Wnt/β‐catenin signaling may also modulate antiviral immunity. Experimental evidence suggests that activation of this pathway can suppress mitochondrial antiviral signaling‐dependent interferon responses through effects on mitochondrial and peroxisomal signaling, a mechanism previously described in several RNA viruses [89].

The Wnt/β‐catenin pathway also intersects with the LIN28B/let‐7 regulatory axis. LIN28B‐mediated repression of let‐7 microRNAs may promote cellular plasticity, stemness‐associated transcriptional programs, and inflammatory signaling. In this context, reduced let‐7 expression reported in patients with moderate‐to‐severe COVID‐19 may reflect broader dysregulation of inflammation and stress‐associated pathways rather than direct oncogenic transformation [91]. Because let‐7 targets multiple oncogenic mediators, including STAT3, RAS, HMGA2, and regulators associated with the Wnt/β‐catenin pathway, disruption of this regulatory network could potentially reinforce inflammatory feed‐forward loops [92].

Collectively, the let‐7‐LIN28B–STAT3–Wnt/β‐catenin axis represents a shared regulatory framework linking inflammatory responses observed during SARS‐CoV‐2 infection with signaling pathways also implicated in cancer biology. Nevertheless, most evidence remains derived from in vitro, transcriptomic, and in vivo studies, and the functional consequences of these alterations in vivo, particularly regarding long term cancer risk, remain speculative.

## 10 MicroRNAs as Integrators of Inflammation, Stemness, Oncogenic Signaling and SARS‐CoV‐2 Infection

MicroRNAs (miRNAs) function as post‐transcriptional regulators that integrate inflammatory, immune, metabolic, and oncogenic signaling pathways [93]. SARS‐CoV‐2 infection has been associated with substantial remodeling of the host miRNA landscape, with several studies reporting expression changes that correlate with disease severity, inflammatory status, and post‐acute manifestations [94, 95]. Importantly, many of these dysregulated miRNAs converge on signaling networks also implicated in cancer biology, including STAT3, NF‐κB, Wnt/β‐catenin, PI3K/AKT, and epithelial–mesenchymal transition (EMT)‐associated pathways [96] (See Figure 2D).

Among the reported alterations, miR‐21 is consistently elevated in acute COVID‐19 and has been associated with disease severity and prolonged intensive care requirements [96]. In oncologic settings, miR‐21 is recognized as a protumorigenic miRNA capable of promoting proliferation, EMT‐associated programs, and metastatic behavior through modulation of tumor suppressor pathways, including LZTFL1 and Wnt/β‐catenin signaling [97, 98]. Similarly, pro‐inflammatory miRNAs such as miR‐155 and the miR‐221/222 cluster have been reported to increase during acute SARS‐CoV‐2 infection [93]. These miRNAs are also implicated in lymphoma biology, solid tumor progression, immune dysregulation, and resistance to endocrine therapies in breast cancer contexts [97, 98, 99]. In addition, miR‐31‐5p has been described as one of the most strongly upregulated miRNAs during early COVID‐19, where it may contribute to inflammatory amplification and cellular plasticity programs previously associated with tumor invasion and EMT‐related signaling [93, 94].

Conversely, reduced expression of tumor suppressor‐associated miRNAs, including members of the let‐7 family, miR‐150‐5p, and miR‐93‐5p, has been linked to impaired immune regulation, stemness‐associated transcriptional programs, and deregulated STAT3/Wnt signaling. In particular, downregulation of let‐7, a known inhibitor of STAT3, RAS, HMGA2, and Wnt/β‐catenin‐associated pathways, emerges as a shared molecular feature reported in severe COVID‐19 and several malignancies, potentially facilitating inflammatory and survival‐associated signaling networks [95] (See Figure 2C). Furthermore, reduced circulating levels of miR‐150‐5p and miR‐93‐5p have been associated with adverse outcomes in cancer patients infected with SARS‐CoV‐2 [95], although whether these changes reflect SARS‐CoV‐2‐specific effects or broader systemic inflammatory stress remains unclear.

Importantly, current evidence does not support a direct oncogenic role for individual miRNAs during SARS‐CoV‐2 infection. Many of the miRNA alterations summarized in Table 1 are not unique to COVID‐19 and may also occur in other inflammatory or infectious conditions. Therefore, these observations should be interpreted as reflecting convergence on shared stress‐response and inflammatory regulatory networks rather than SARS‐CoV‐2‐specific oncogenic signatures. In addition, most available evidence derives from transcriptomic analyses, plasma or serum profiling studies, and relatively small patient cohorts, while the persistence and functional consequences of these alterations in vivo remain incompletely understood. Nevertheless, the overlap between COVID‐19‐associated miRNA dysregulation and pathways implicated in inflammation, immune evasion, cellular plasticity, and tumor progression supports their relevance as candidate mechanistic mediators and biomarkers deserving further longitudinal investigation.

**TABLE 1:** Summary of cancer‐relevant miRNAs dysregulated during SARS‐CoV‐2 infection.

miRNA	Dysregulation in COVID‐19	Related oncological process	Sample tissue	Sample size (*N*)	Detection method	Statistical significance (p)	Variation (fold change)	Supporting study
*miR‐21*	↑	Oncogenic miRNA; promotes proliferation, EMT, and immune evasion by targeting LZTFL1.	Serum/Plasma	Garg: 18 ICU cases, 15 HC; Wang: 252 Breast Cancer	RT‐qPCR	≤ 0.05 (acute cases)	Elevated in acute phase	[96, 97, 98]
*let‐7 family (a, b, c)*	↓	Tumor suppressor; inhibits stemness, EMT, and inflammation via the let‐7–STAT3–Wnt axis.	Saliva and Plasma	10 MD, 10 SD, 10 HC	qPCR array	< 0.05	2.5 fold regulation	[64, 94]
*miR‐31‐5p*	↑	Promotes tumor invasion and inflammatory signaling; suppresses Wnt antagonists.	Plasma	10 COVID‐19, 10 HC	NGS and qRT‐PCR	< 0.05	50‐fold upregulation	[93]
*miR‐150‐5p*	↓	Tumor suppressor; critical immune surveillance regulator; inhibits viral nsp10.	Plasma	128 Cancer patients with COVID‐19	RT‐qPCR	< 0.0001	Significant decrease linked to death	[95]
*miR‐93‐5p*	↓	Regulates systemic inflammation and Th17 cell differentiation via STAT3.	Plasma	128 Cancer patients with COVID‐19	RT‐qPCR	0.0017	Significant decrease linked to death	[95]
*miR‐155*	↑	Pro‐inflammatory; linked to lymphomas, solid tumors, and therapy resistance.	Plasma	10 COVID‐19	NGS/RT‐qPCR	< 0.05	Elevated in acute phase	[93, 99]
*miR‐221/222*	↑	Promotes cell cycle progression (p27/PTEN) and endocrine therapy resistance.	Plasma	10 COVID‐19	NGS/RT‐qPCR	< 0.05	Elevated in acute phase	[93, 99]

*Note:* Comprehensive overview of miRNAs significantly altered in response to SARS‐CoV‐2 infection and their documented involvement in tumor biology. It details the direction of dysregulation (↑ upregulation or ↓ downregulation) and associated oncological processes, including proliferative signaling, EMT, and immune surveillance. The data integrate clinical parameters such as sample tissue origin, sample size, detection methodology, statistical significance, and fold change variation.

Abbreviations: EMT: epithelial‐mesenchymal transition; HC: healthy controls; MD: mild disease; NGS: next‐generation sequencing; SD: severe disease.

## 11 Impact of the COVID‐19 Pandemic on Cancer Incidence and Stage at Diagnosis: Clinical and Biological Implications

The COVID‐19 pandemic has had a profound and multifaceted impact on cancer detection, diagnosis, and clinical outcomes. During 2020, widespread suspension of screening programs, reduced access to primary care, and delays in diagnostic procedures led to a marked decline in newly diagnosed cancer cases across multiple healthcare systems worldwide [6]. Registry‐based and population‐level studies consistently reported sharp reductions in cancer incidence during the early pandemic period, followed by a partial and heterogeneous recovery in subsequent years [6].

Importantly, accumulating evidence indicates that this transient decline in cancer diagnoses does not reflect a true reduction in cancer burden, but rather delayed detection. Several studies have documented a shift toward more advanced stages at diagnosis and an increased proportion of metastatic presentations following the acute phases of the pandemic [100].

Beyond healthcare system disruptions, the biological consequences of SARS‐CoV‐2 infection warrant consideration when interpreting post‐pandemic cancer trends. As outlined in this review, SARS‐CoV‐2 induces persistent inflammatory, immunological, and epigenetic alterations that overlap with core mechanisms of tumor initiation and progression. Chronic inflammation, immune suppression, cellular senescence, metabolic reprogramming, and dysregulation of tumor‐suppressive microRNAs, particularly within the let‐7–STAT3–Wnt axis, represent convergent pathways through which viral infection could plausibly influence cancer risk or disease behavior in susceptible individuals [67].

At present, epidemiological evidence directly linking SARS‐CoV‐2 infection to increased cancer incidence remains limited and inconclusive. Large observational cohorts have suggested possible associations for selected cancer types, including virally associated malignancies; however, these findings are subject to confounding, surveillance bias, and insufficient follow‐up duration [101]. Consequently, SARS‐CoV‐2 cannot be classified as an oncogenic virus based on current data.

Nevertheless, the convergence of indirect clinical effects and biologically plausible mechanisms underscores the importance of long‐term surveillance of COVID‐19 survivors, particularly those with preexisting malignancies, premalignant conditions, or known oncogenic risk factors. Integrating mechanistic insights with longitudinal clinical and epidemiological studies will be essential to disentangle the relative contributions of healthcare disruption and post‐infectious biology to future cancer burden.

## 12 Perspectives

In conclusion, the COVID‐19 pandemic has profoundly reshaped cancer care worldwide, while its potential long‐term implications for cancer biology remain incompletely defined. Current evidence indicates that SARS‐CoV‐2 infection may induce persistent inflammation, immune dysregulation, metabolic stress, and epigenetic alterations that overlap with pathways involved in cancer biology, although direct causality has not been established. These effects may be particularly relevant in subsets of individuals with post‐acute sequelae of SARS‐CoV‐2 infection (Long COVID), in whom inflammatory and immune abnormalities may persist for months after the acute phase.

Among the proposed mechanisms, the strongest evidence currently supports persistent inflammation and immune dysregulation, including chronic IL‐6/STAT3 and NF‐κB activation, cytokine imbalance, T‐cell exhaustion, and impaired immune surveillance, as these alterations have been consistently documented in severe COVID‐19 and subsets of Long COVID patients. Senescence‐associated inflammatory signaling, oxidative stress, mitochondrial dysfunction, and metabolic dysregulation also represent biologically plausible contributors supported by experimental models and selected human tissue studies. In contrast, perturbation of tumor suppressor pathways such as p53 and pRB is supported primarily by mechanistic and in vitro studies, while direct oncogenic or transformation‐like mechanisms remain highly speculative and currently lack convincing clinical evidence.

Future longitudinal studies should integrate immune profiling, inflammatory biomarkers, and transcriptomic or epigenetic signatures with long‐term clinical outcomes, particularly cancer incidence, recurrence, and progression. Persistent IL‐6/STAT3 and NF‐κB activation, senescence‐associated inflammatory programs, and immune exhaustion phenotypes may represent especially informative pathways for longitudinal monitoring.

Importantly, current evidence primarily supports the convergence of inflammatory, immune, metabolic, and stress‐response pathways shared between SARS‐CoV‐2 infection and cancer biology, rather than direct oncogenic transformation by the virus itself. In this context, the most relevant question may not be whether SARS‐CoV‐2 acts as a classical oncogenic virus, but whether persistent post‐infectious immune and inflammatory alterations create biological conditions capable of modulating tumor progression, cellular plasticity, or reactivation of pre‐existing malignant states in susceptible individuals. New interdisciplinary and longitudinal research will be essential to clarify the relative contributions of persistent inflammation, host susceptibility, and pre‐existing oncogenic conditions to the evolving cancer landscape in the post‐pandemic era.

## Author Contributions

**Alejandro Cornejo:** writing – original draft, writing – review and editing, visualization, software. **Andrea Orue:** conceptualization, investigation, writing – original draft, data curation, supervision, writing – review and editing, formal analysis. **Héctor Rafael Rangel:** conceptualization, writing – original draft, writing – review and editing, formal analysis, data curation.

## Funding

The authors have nothing to report.

## Conflicts of Interest

The authors declare no conflicts of interest.

## Data Availability

Data sharing is not applicable to this article as no new datasets were generated or analyzed during the current study. All data supporting the theoretical and potential mechanisms discussed herein are derived from peer‐reviewed, publicly available literature cited within the manuscript.
